# miRNAs in Signal Transduction of SMAD Proteins in Breast Cancer

**DOI:** 10.3390/ijms251810088

**Published:** 2024-09-19

**Authors:** Tomasz Sirek, Agata Sirek, Przemysław Borawski, Nikola Zmarzły, Joanna Sułkowska, Katarzyna Król-Jatręga, Marcin Opławski, Dariusz Boroń, Michał Chalcarz, Piotr Ossowski, Konrad Dziobek, Damian Strojny, Kacper Boroń, Dominika Janiszewska-Bil, Beniamin Oskar Grabarek

**Affiliations:** 1 Department of Plastic and Reconstructive Surgery, Hospital for Minimally Invasive and Reconstructive Surgery in Bielsko-Biała, 43-316 Bielsko-Biala, Poland; agatasirek85@gmail.com (A.S.); katarzynakroljatrenga@gmail.com (K.K.-J.); 2 Department of Medical and Health Sciences, Collegium Medicum, WSB University, 41-300 Dabrowa Górnicza, Poland; nikola.zmarzly@gmail.com (N.Z.); joanna.sulkowska@wsb.edu.pl (J.S.); dariusz.boron@gmail.com (D.B.); chalcarzmichal@gmail.com (M.C.); drpiotrossowski@gmail.com (P.O.); konraddziobek28@gmail.com (K.D.); strojny.ds@gmail.com (D.S.); q375@icloud.com (K.B.); dominika.bjaniszewska@gmail.com (D.J.-B.); bgrabarek7@gmail.com (B.O.G.); 3 Independent Researcher, 87-800 Włocławek, Poland; przembor3@gmail.com; 4 Department of Gynecology and Obstetrics with Gynecologic Oncology, Ludwik Rydygier Memorial Specialized Hospital, 31-826 Kraków, Poland; marcin.oplawski@gmail.com; 5 Department of Gynecology and Obstetrics, Faculty of Medicine and Health Sciences, Andrzej Frycz Modrzewski University in Cracow, 30-705 Kraków, Poland; 6 Institute of Clinical Science, Skłodowska-Curie Medical University, 00-136 Warszawa, Poland; 7 Department of Gynecology and Obstetrics, TOMMED Specjalisci od Zdrowia, 40-662 Katowice, Poland; 8 Chalcarz Clinic-Aesthetic Surgery, Aesthetic Medicine, 60-001 Poznan, Poland; 9 Bieńkowski Medical Center-Plastic Surgery, 85-020 Bydgoszcz, Poland; 10 Institute of Health Care, National Academy of Applied Sciences in Przemyśl, 37-700 Przemyśl, Poland; 11 New Medical Techniques Specjalist Hospital of St. Family in Rudna Mała, 36-054 Rudna Mala, Poland; 12 Department of Molecular, Biology Gyncentrum Fertility Clinic, 40-055 Katowice, Poland

**Keywords:** breast cancer, miRNA, SMAD, transforming growth factor beta

## Abstract

The aim of this study was to identify miRNAs that could potentially influence the activity of SMAD proteins involved in TGFβ signal transduction in five types of breast cancer in Polish women. Patients with five breast cancer subtypes were included in the study: luminal A (*n* = 130), luminal B HER2− (*n* = 100), luminal B HER2+ (*n* = 96), non-luminal HER2+ (*n* = 36), and TNBC (*n* = 43). During surgery, tumor tissue was removed along with a margin of healthy tissue (control). Molecular analysis included determination of the expression of genes related to SMAD protein signal transduction using mRNA microarrays and reverse transcription quantitative polymerase chain reaction (RT-qPCR). Protein expression was determined using an enzyme-linked immunosorbent assay (ELISA). The miRNA profiling was performed using miRNA microarrays and the miRDB database. SMAD3 and SMAD5 were overexpressed in all types of breast cancer, which could be related to the reduced expression of miR-145, and the findings for SMAD4 and miR-155 were similar. Additionally, the level of SMAD7 was reduced, which may be due to the low activity of miR-15b and miR21b. This study determined the gene expression profiles involved in SMAD protein signal transduction across five different types of breast cancer and identified the miRNAs potentially regulating their activity. Overexpression of SMAD3, SMAD4, and SMAD5 suggests excessive activation of the TGFβ pathway, potentially promoting tumor growth and development. Concurrently, a significant reduction in SMAD7 expression removes inhibitory control in the TGFβ pathway, a phenomenon that is particularly evident in more aggressive breast cancer types.

## 1. Introduction

Breast cancer is the most frequently diagnosed malignancy in women around the world. According to data from the World Health Organization (WHO), in 2020, it accounted for approximately 25% of all diagnosed cancers. Moreover, breast cancer accounted for approximately 16% of cancer-related deaths in women [1]. Data from the National Cancer Registry in Poland indicate disturbing trends. Breast cancer is the leading cause of death in women under 65 years of age, including approximately 30% of women aged 20 to 44 and over 40% of women aged 45 to 65. In young women, this cancer is dominant both in terms of morbidity and mortality [2].

Breast cancer shows significant differences in molecular profiles and clinicopathological features. For this reason, its treatment is complicated and requires a combination of various methods, including surgery, chemotherapy, radiotherapy, hormonal therapy, and targeted therapies [3]. The classification of the tumor is based on the activity of the estrogen receptor (ER), progesterone receptor (PR), human epidermal growth factor receptor 2 (HER2), and the Ki67 proliferation index. As a result, it is possible to distinguish the main subtypes: luminal A, luminal B, HER2-positive, and triple-negative breast cancer (TNBC) [4]. The luminal A subtype (characterized by ER+, PR+, HER2−, and low levels of Ki-67) accounts for the largest percentage of breast cancers. It usually has a good prognosis due to its slow growth and less aggressive nature [3]. The luminal B subtype is characterized by heightened activity of genes related to proliferation and a worse prognosis compared to the luminal A subtype. The presence of ER is observed, and HER2 may or may not be present [5]. The non-luminal HER2-positive subtype is characterized by the overexpression of HER2+ with downregulation of ER and PR. This is associated with increased aggressiveness of the tumor due to intensified cell proliferation and division [6]. Triple-negative breast cancer (TNBC), which is characterized by ER−, PR−, and HER2−, is more aggressive compared to other subtypes. It also has a worse prognosis [7].

The TGFβ pathway participates in the regulation of many important processes, including cell proliferation, apoptosis, and differentiation. This pathway may also contribute to the induction of tumorigenesis processes, including the promotion of invasion or epithelial–mesenchymal transition (EMT) [8]. SMAD (mothers against decapentaplegic homolog) proteins participate in signal transduction of the transforming growth factor beta (TGFβ) pathway. SMAD 1/2/3/5/8 are proteins that are activated by the receptor and are phosphorylated in response to activation of the TGFβ pathway [9]. They then form a complex with the SMAD4 protein, which enables its transport from the cytoplasm to the cell nucleus. Transcription factors are activated, which leads to the inhibition of E-cadherin expression and an increase in the activity of genes characteristic of the mesenchymal phenotype [10]. SMAD6 and SMAD7 are involved in the negative regulation of signaling by binding to the TGFβ receptor [11] (Figure S1). 

Research conducted to date indicates the significant importance of impaired functioning of SMAD proteins in the development of breast cancer. Knockdown of SMAD3 can inhibit tumorigenesis in a mouse model [9] and delay the occurrence of bone metastasis [12] and EMT in cell lines [13]. In addition, depending on the expression of SMAD proteins, resistance to different drugs may be observed [9]. 

MiRNA molecules are short, non-coding, single-stranded RNAs that participate in the regulation of the expression of approximately 60% of human genes [14]. This regulation is carried out at the post-transcriptional level by binding to the target mRNA. Depending on the function of the gene, miRNAs may have both suppressor and oncogenic functions [15]. Importantly, a single miRNA can participate in the regulation of the activity of multiple genes. At the same time, several different miRNAs can simultaneously control the action of a single gene. Although the miRNA network is complex, it is also the subject of many studies because the discovery of miRNAs and their targets can help in the diagnosis and discovery of new therapeutic targets [16]. Previous studies have shown that miRNA molecules may be important in the development of breast cancer. They can enhance invasion and metastasis by targeting tumor suppressor genes, whereas miRNAs important to maintaining physiological state are downregulated [17]. As a result, the mechanisms of breast cancer formation may depend on many factors, their mutual relations, and the biological context.

The aim of this study was to determine the miRNAs that could potentially influence the activity of SMAD proteins involved in TGFβ signal transduction in five types of breast cancer in Polish women.

## 2. Results

The analysis included 405 patients divided into five groups according to the molecular subtype of breast cancer: luminal A (*n* = 130), luminal B HER2-negative (*n* = 100), luminal B HER2-positive (*n* = 96), HER2-positive (*n* = 36), and triple-negative breast cancer (TNBC). Analysis at the mRNA level was performed using microarrays and RT-qPCR. Then, the level of selected proteins was determined using ELISA. The last step was to identify differential miRNAs using microarrays and search for their targets among the selected genes using the miRDB database.

### 2.1. Gene Expression Profile Determined by mRNA Microarrays

Microarray analysis was conducted to comprehensively profile gene expression changes in different breast cancer subtypes, allowing us to identify differentially expressed SMAD proteins involved in TGFβ signal transduction.

The overrepresentation test identified 12 genes associated with SMAD protein signal transduction out of 86 genes involved in TGFβ signaling (Table 1).

The identified genes included SMADs, growth differentiation factors, inhibin, and TGFβ receptor 2. These genes corresponded to 35 mRNAs on the microarray. The analysis showed that, of these 35 mRNAs, 7 mRNAs significantly changed their expression in each type of breast cancer compared to the control (*p* < 0.05; FC > 2 or <−2). Detailed results are included in Table 2.

The analysis showed that mRNAs representing *SMAD3*, *SMAD4*, and *SMAD5* exhibit overexpression, reaching the highest level in TNBC. In the case of *SMAD7*, a significant decrease in its expression was noted, and it was also most pronounced in TNBC.

### 2.2. Expression Profile of SMAD3, SMAD4, SMAD5, and SMAD7 Determined by RT-qPCR and ELISA

The microarray experiment was then validated by RT-qPCR to ensure the accuracy of the microarray results. Four differential genes selected in the microarray analysis were included in the analysis: *SMAD3*, *SMAD4*, *SMAD5*, and *SMAD7*. Table 3 shows the detailed results.

The results indicate overexpression of *SMAD3*, *SMAD4*, and *SMAD5* with reduced levels of *SMAD7*, which is consistent with the microarray experiment.

The expression of the analyzed genes was then determined at the protein level to confirm whether the transcriptional changes observed via microarray analysis and RT-qPCR translated into differences in protein expression (Table 4).

The analysis showed a significant increase in the concentration of SMAD3, SMAD4, and SMAD5 in all types of breast cancer samples compared to the control. In turn, the concentration of SMAD7 decreased significantly, reaching a level below detection in the luminal B HER2−, luminal B HER2+, non-luminal HER2+, and TNBC groups. In addition, the protein concentration in these groups changed significantly compared to the luminal A group.

### 2.3. miRNA Target Prediction

In the last step, miRNA microarray analysis was performed to identify differentially expressed miRNAs in each breast cancer subtype, and then it was determined whether they could participate in the regulation of the expression of *SMAD3*, *SMAD4*, *SMAD5*, and *SMAD7* selected in previous steps (Table 5).

It was observed that low miR-155 expression may be related to the overexpression of *SMAD4* (with a target score of 83). Moreover, high expression of *SMAD3* and *SMAD5* may be a consequence of a decrease in miR-155 activity, which showed target scores of 98 and 93 for these genes, respectively. The analysis also showed that reduced *SMAD7* expression may result from increased miR-15b and miR-21 activity (both showing the same target score of 92).

## 3. Discussion

In this study, we determined the expression levels of genes associated with signal transduction from the TGFβ pathway by SMAD proteins. The analysis at the mRNA level showed increased expression of *SMAD3-5* and decreased levels of *SMAD7*. This result was confirmed at the protein level. In addition, predictions were made regarding which miRNAs differentiating breast cancer from the control may participate in the regulation of SMAD protein activity.

The SMAD2 and SMAD3 are important downstream proteins of the TGFβ pathway. Despite their close relationship, previous research indicates that SMAD3 plays an important role in TGFβ signal transduction. Singa et al. reported that the knockdown of SMAD3 in TNBC target cell lines reduced the invasive potential of breast cancer, whereas the knockdown of SMAD2 did not have this effect [18]. In turn, Petersen et al. showed that significant genes associated with bone metastasis depend on the level of SMAD3, but not SMAD2. Interestingly, SMAD2 knockdown resulted in increased aggressiveness in MDA-MB-231 cells [12]. Chen et al. observed a reduction in SMAD3 and SMAD7 expression in stage II breast cancer. In turn, in the xenograft tumor mouse model, SMAD3 knockdown inhibited tumor formation [9]. Yang et al. demonstrated that SMAD3 can be targeted by miR-135-5p, which leads to TGFβ/SMAD signaling inhibition [19]. Interestingly, our study identified another miRNA that may be involved in the regulation of *SMAD3* activity. The miRNA prediction in our study revealed a potential association between SMAD3 and miR-145, which is consistent with the results of previous studies [20,21]. MiR-145 inhibited invasion and metastasis in breast cancer [21], but also in colorectal cancer [22] and non-small cell lung cancer [23]. Manvati et al. showed that miR-145 reduced SMAD3 activity in the MCF7 breast cancer cell line [20]. Our study showed overexpression of SMAD3 in all five types of breast cancer, which may be related to a significant reduction in miR-145 activity. Moreover, this relationship deepened as tumor aggressiveness increased. Our study also identified an association between miRNA-145 and SMAD5. Opyrchal et al. showed that SMAD5 expression is needed to promote chemoresistance in breast cancer [24]. In other cancers, high SMAD5 levels promoted poorer overall survival in gastric cancer, shorter survival in lymphatic leukemia, and bladder cancer progression [25]. In our study, the highest overexpression of this gene was observed in TNBC, which was accompanied by the largest decrease in miRNA-145 activity compared to other types of breast cancer.

During miRNA prediction, miR-155 was identified, with its reduced activity potentially associated with SMAD4 overexpression. Wang et al. observed that miR-155 overexpression could inhibit tumor progression and metastasis. For this reason, the detection of this miRNA in serum may indicate a good prognosis, and increasing its expression in the tumor may improve the effectiveness of immune checkpoint blockade therapy [26].

SMAD7 is an important regulatory element in the signal transduction of the TGFβ pathway. It can act both at the cytoplasmic and nuclear levels by blocking the activation of regulatory SMADs, inducing the degradation of the TGFβ receptor, or disrupting the formation of SMAD complexes and their binding to DNA. In addition to the TGFβ pathway, it can promote tumor necrosis factor-related apoptosis and also interact with the nuclear factor kappa-light-chain-enhancer of activated B cells (NF-kB) pathway by lowering the expression of pro-inflammatory genes [27]. Ryu et al. observed that increased SMAD7 levels in breast cancer inhibited metastasis [28]. Moreover, Smith et al. showed that the miR-106b-25 cluster is involved in the regulation of SMAD7 activity. Decreased SMAD7 levels were associated with excessive stimulation of TGFB signaling and induction of EMT [29].

In our study, SMAD7 expression significantly decreased in all types of breast cancer, with the greatest decrease observed in TNBC. This may indicate the loss of inhibition of the TGFβ pathway, leading to its excessive activity, which is particularly visible in more aggressive breast cancers. Our study showed a potential association between reduced SMAD7 expression and the overexpression of miR-15b and miR-21b. Qi et al. reported that miR-15b can stimulate the migration and invasion of breast cancer cells [30]. Wu et al. observed a similar effect on the MCF-7 and MD-MBA-231 cell lines and xenograft mouse model [31]. In the case of miR-21b, its overexpression was noted, which is consistent with our observations [32]. Interestingly, Wang et al. demonstrated that miR-21 promotes proliferation and metastasis in breast cancer [33].

## 4. Materials and Methods

### 4.1. Patients

The study enrolled 405 patients diagnosed with distinct breast cancer subtypes: luminal A (*n* = 130), luminal B (HER2-negative (*n* = 100) and HER2-positive (*n* = 96) subgroups), HER2-positive (*n* = 36), and triple-negative breast cancer (TNBC) (*n* = 43). The patients underwent surgery, during which the cancerous tissue was removed along with a margin of healthy tissue. Based on pathological evaluation during the surgery, a distinction was made between tumor-affected tissue (study groups) and tumor-free tissue (control). All patients were classified according to the tumor, nodule, and metastasis (TNM) staging system as T1N0M0. Detailed patient characteristics are presented in Table 6.

The study was carried out in accordance with the 2013 Helsinki Declaration and was approved by the Bioethical Committee of the Regional Medical Chamber in Krakow on 10 March 2023 (81/KBL/OIL/2023). Informed consent was obtained from all patients.

### 4.2. Total Ribonucleic Acid (RNA) Extraction

Total RNA extraction from tissues was performed using the TRIzol reagent (Invitrogen Life Technologies, Carlsbad, CA, USA; cat. no. 15596026) according to the manufacturer’s protocol. An RNeasy mini kit (QIAGEN, Hilden, Germany; cat. no. 74104) and DNase I (Fermentas International Inc., Burlington, ON, Canada; cat. no. 18047019) were used to purify the obtained extracts. The extracted RNA was assessed qualitatively and quantitatively by 1% agarose gel electrophoresis and absorbance measurement.

### 4.3. mRNA Microarray Analysis

The expression profile was assessed using HG-U133A 2_0 oligonucleotide microarrays (Affymetrix, Santa Clara, CA, USA) and the GeneChip™ 3′IVT PLUS kit (Thermo Fisher Scientific, Inc., Waltham, MA, USA; cat. No. 902416) according to the manufacturer’s instructions. The list of 86 genes that participate in TGFβ signaling was prepared according to the Kyoto Encyclopedia of genes and genomes (KEGG) pathway map (hsa04350). A binomial overrepresentation test with Bonferroni correction was performed using the Protein Analysis Through Evolutionary Relationship (PANTHER) tool. From the list of GO Biological Processes, “SMAD protein signal transduction” was selected [34].

### 4.4. Reverse Transcription Quantitative Polymerase Chain Reaction (RT-qPCR)

RT-qPCR was performed to confirm the results of the microarray experiment. The SensiFast SYBR No-ROX One-Step Kit (Bioline, London, UK) was used to assess the expression profile of selected genes in accordance with the manufacturer’s recommendations. β-actin (ACTB) was utilized as the endogenous control. Expression profiles were determined using the 2^−ΔΔCt^ method.

The thermal profile included reverse transcription (45 °C, 10 min), polymerase activation (95 °C, 2 min), and 40 cycles of denaturation (95 °C, 5 s), annealing (60 °C, 10 s), and elongation (72 °C, 5 s). The primer sequences are listed in Table 7.

### 4.5. miRNA Profiling

The miRNA 2.0 Microarrays (Affymetrix, Santa Clara, CA, USA), FlashTag Biotin HSR RNA Labeling Kit (Affymetrix, Santa Clara, CA, USA), and Hybridization Wash and Stain Kit (Affymetrix, Santa Clara, CA, USA) were used as recommended by the manufacturer. The mirDB tool (http://mirdb.org, accessed on 17 June 2024) was then used to predict miRNA targets among the selected genes (target score ≥ 80) [35]. 

### 4.6. Enzyme-Linked Immunosorbent Assay (ELISA)

The protein expression profile was determined by ELISA (Abbexa, Cambridge, UK) using the following ELISA kits: Human Mothers Against Decapentaplegic Homolog 3 (Smad3) Kit (MyBioSource, Inc., San Diego, CA, USA; cat. no. MBS161553), Human Mothers Against Decapentaplegic Homolog 4 (Smad4) Kit (MyBioSource, Inc., San Diego, CA, USA; cat. no. MBS450115), Human Mothers against decapentaplegic homolog 5 Smad5 Kit (MyBioSource, Inc., San Diego, CA, USA; cat. no. MBS1608900), and Human Mothers against decapentaplegic homolog 7, Smad7 Kit (MyBioSource, Inc., San Diego, CA, USA; cat. no. MBS162196). The sample preparation followed the manufacturer’s protocol. One hundred milligrams of tissue was rinsed with PBS and homogenized in approximately 1 mL of PBS on ice. Samples were frozen at −20 °C and then thawed at 2–8 °C. Finally, the homogenates were centrifuged for 15 min at 10,000 rpm at 4 °C. The supernatant was collected for the assay.

### 4.7. Statistical Analysis

The Transcriptome Analysis Console software 2.0 (Thermo Fisher Scientific, Waltham, MA, USA) was used to analyze the results of the microarray experiments. One-way ANOVA and Tukey’s post hoc test were performed (*p* < 0.05; FC > 2 or FC < −2). The analyses of RT-qPCR and ELISA results were performed using the Statistica 13.3 (StatSoft, Kraków, Poland). Lack of normality in data distribution was confirmed by the Shapiro–Wilk test. Then, ANOVA Kruskal–Wallis and Dunn’s tests were performed.

The group size was estimated using a sampling calculator [36]. The recommended number of participants for the study was 377, assuming a confidence level of 95%, a margin of error of 5%, and a total of approximately 19,620 women diagnosed with breast cancer in Poland in 2019 [37].

## 5. Conclusions

This study allowed us to determine the expression profile of genes involved in SMAD protein signal transduction in five different types of breast cancer, as well as to identify miRNAs potentially related to the regulation of their activity. Overexpression of SMAD3, SMAD4, and SMAD5 indicates excessive stimulation of the TGFβ pathway, which may promote tumor proliferation and development. This may be related to the lack of regulatory influence of miR-145 and miR-155, the activity of which was reduced. At the same time, a significant decrease in SMAD7 expression removes regulatory breaks in the TGFβ pathway, which is particularly evident in more aggressive types of breast cancer. The present study identified potential relationships between SMAD proteins and miRNAs in various types of breast cancer, which may be used in further research on targeted therapy. Several methods were used to determine and validate the expression profile at both the mRNA and protein levels. A limitation of our study is the smaller non-luminal HER2+ and TNBC groups compared to the other groups. Furthermore, the study recruited Polish women, which limits the diversity of the patient cohort and may limit the broader applicability of our results. In addition, we took a broader approach, looking at the subtypes as a whole to detect general patterns, which may have obscured differences between specific grades and age groups. It would be beneficial to expand the research with additional methods, including PCR for miRNAs, and involve more detailed comparisons of grades and age groups to refine our findings.

## Figures and Tables

**Table 1:** List of genes involved in SMAD protein signal transduction.

Gene Symbol	Fold Enrichment	*p*-Value
*GDF5*, *GDF6*, *INHBA*, *SMAD1*, *SMAD2*, *SMAD3*, *SMAD4*, *SMAD5*, *SMAD6*, *SMAD7*, *SMAD9*, *TGFBR2*	>100	<0.0001

GDF—growth differentiation factor; INHBA—inhibin beta A chain; SMAD—mothers against decapentaplegic homolog; TGFBR2—transforming growth factor beta receptor type-2.

**Table 2:** List of mRNAs representing genes involved in SMAD protein signal transduction differentiating breast cancer from the control regardless of its type (*p* < 0.05; FC > 2 or <−2).

ID	mRNA	Fold Change				
		LumA vs. C	LumB HER2− vs. C	LumB HER2+ vs. C	Non-Luminal HER2+ vs. C	TNBC
218284_at	*SMAD3*	2.29	2.56	2.81	2.69	4.22
202527_s_at 235725_at	*SMAD4*	2.05 2.12	2.48 3.17	3.34 2.49	3.04 3.90	4.01 4.38
205188_s_at 235451_at	*SMAD5*	2.08 2.04	2.32 2.12	2.32 2.17	2.97 3.68	3.15 3.72
204790_at	*SMAD7*	−3.43	−3.75	−3.86	−3.66	−4.63

ID—number of the probe; LumA—luminal A; LumB—luminal B; HER2—human epidermal growth factor receptor 2; TNBC—triple-negative breast cancer; C—control; SMAD—mothers against decapentaplegic homolog.

**Table 3:** Expression profile of *SMAD3*, *SMAD4*, *SMAD5*, and *SMAD7* determined by RT-qPCR (*p* < 0.05).

mRNA	Fold Change				
	LumA vs. C	LumB HER2− vs. C	LumB HER2+ vs. C	Non-Luminal HER2+ vs. C	TNBC
SMAD3	2.99	3.64	4.22	3.96	6.13
SMAD4	3.35	3.95	4.45	5.09	4.82
SMAD5	3.88	3.97	3.73	4.27	4.38
SMAD7	−6.15	−7.33	−8.45	−8.58	−12.02

LumA—luminal A; LumB—luminal B; HER2—human epidermal growth factor receptor 2; TNBC—triple-negative breast cancer; C—control; SMAD—mothers against decapentaplegic homolog.

**Table 4:** Concentration of SMAD3, SMAD4, SMAD5, and SMAD7 in breast cancer and control groups (*p* < 0.05).

Protein [ng/mL]	Control	Luminal A	Luminal B HER2−	Luminal B HER2+	Non-Luminal HER2+	TNBC
SMAD3	1.82 ± 0.13	3.15 ± 0.13 *	3.82 ± 0.14 *	4.21 ± 0.13 *	4.65 ± 0.11 *	5.38 ± 0.11 *
SMAD4	1.16 ± 0.08	1.34 ± 0.06 *	1.76 ± 0.06 *	1.82 ± 0.05 *	1.91 ± 0.08 *	4.37 ± 0.07 *
SMAD5	0.93 ± 0.06	1.51 ± 0.09 *	1.84 ± 0.1 *	1.85 ± 0.05 *	1.99 ± 0.09 *	3.87 ± 0.1 *
SMAD7	1.38 ± 0.16	0.48 ± 0.04 *	below detection threshold *	below detection threshold *	below detection threshold *	below detection threshold *

HER2—human epidermal growth factor receptor 2; TNBC—triple-negative breast cancer; C—control; SMAD—mothers against decapentaplegic homolog. * *p* < 0.05 vs. control.

**Table 5:** Expression of miRNAs potentially involved in the regulation of the studied genes (*p* < 0.05; FC > 2 or <−2).

mRNA	miRNA	Target Score	Fold Change				
			LumA vs. C	LumB HER2− vs. C	LumB HER2+ vs. C	Non-Luminal HER2+ vs. C	TNBC
*SMAD4*	miR-155	83	−2.02	−2.12	−2.07	−2.77	−5.99
*SMAD3* *SMAD5*	miR-145	98 93	−2.17	−2.01	−2.54	−2.81	−3.7
*SMAD7*	miR-15b	92	2.27	2.42	2.49	2.92	4.66
	miR-21b	92	2.16	2.70	2.67	2.55	4.19

LumA—luminal A; LumB—luminal B; HER2—human epidermal growth factor receptor 2; TNBC—triple-negative breast cancer; C—control; SMAD—mothers against decapentaplegic homolog.

**Table 6:** Characteristics of patients.

Molecular Type	Grade			Age		BMI [kg/m^2^]
	G1	G2	G3	<50 Years	>50 Years	
Luminal A	23 (18%)	48 (37%)	59 (45%)	43 (33%)	87 (67%)	30.78 ± 2.76
Luminal B HER2−	31 (31%)	57 (57%)	12 (12%)	32 (32%)	68 (68%)	30.18 ± 4.56
Luminal B HER2+	23 (24%)	57 (59%)	16 (17%)	19 (20%)	77 (80%)	32.09 ± 6.19
Non-luminal HER2+	9 (25%)	12 (33%)	15 (42%)	9 (25%)	27 (75%)	33.18 ± 5.67
TNBC	14 (32%)	21 (49%)	8 (19%)	10 (23%)	33 (77%)	34.67 ± 2.98

HER2—human epidermal growth factor receptor 2; TNBC—triple-negative breast cancer; BMI—body mass index.

**Table 7:** RT-qPCR primers.

mRNA	RT-qPCR Amplification Primers (5′-3′)
*SMAD3*	Forward: CTACCAGAGAGTAGAGACAC Reverse: TCTCTGGAATATTGCTCTGG
*SMAD4*	Forward: AAAGGTCTTTGATTTGCGTC Reverse: CTATTCCACCTACTGATCCTG
*SMAD5*	Forward: CCAGTCTTACCTCCAGTATTAG Reverse: TCCTAAACTGAACCAGAAGG
*SMAD7*	Forward: CAGATTCCCAACTTCTTCTG Reverse: CTCTTGTTGTCCGAATTGAG
*ACTB*	Forward: TCACCCACACTGTGCCCATCTACGA Reverse: CAGCGGAACCGCTCATTGCCAATGG

SMAD—mothers against decapentaplegic homolog; ACTB—β-actin.

## Data Availability

The data used to support findings of this study are included in this article. The data will not be shared due to third-party rights and commercial confidentiality.

## Supplementary Materials

The following supporting information can be downloaded at https://www.mdpi.com/article/10.3390/ijms251810088/s1.
