# Supplementary figures and images for: miRNAs in Signal Transduction of SMAD Proteins in Breast Cancer
Source: Int J Mol Sci. 2024 Sep 19;25(18):10088. doi: 10.3390/ijms251810088 (PMC11432703; doi:10.3390/ijms251810088)

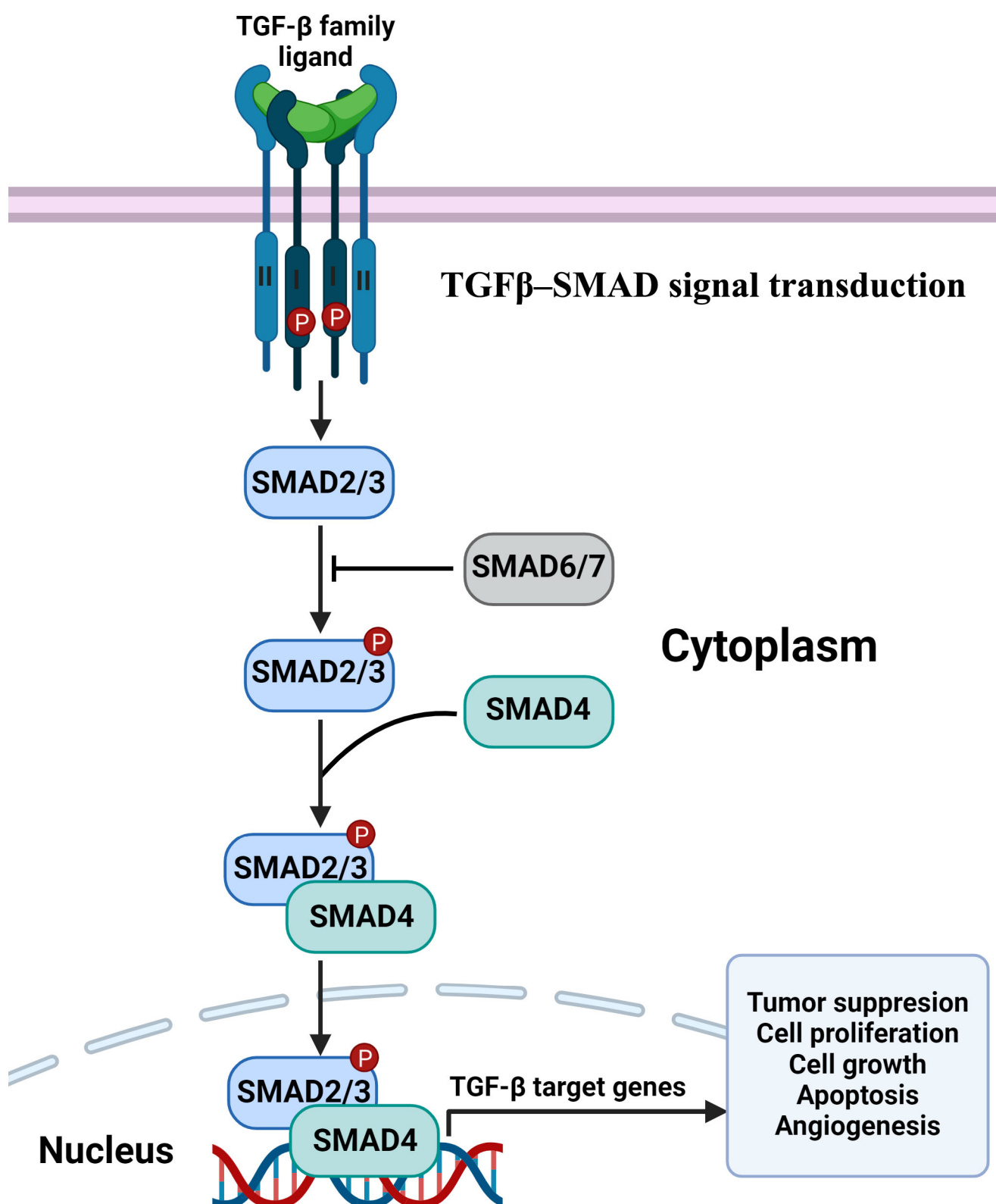

**Figure S1.** Involvement of SMADs in TGF $\beta$  signal transduction.

Supplement: Supplementary file 1 [file ijms-25-10088-s001.zip › ijms-3185471-supplementary.pdf]
